# Supplementary material for: Development of auditory and spontaneous movement responses to music over the first postnatal year
Source: eLife. 2026 Jul 7;14:RP107088. doi: 10.7554/eLife.107088 (PMC13341111; doi:10.7554/eLife.107088)
Supplement: Supplementary file 1. [file elife-107088-supp1.docx]

*Table S1*. Continued acoustic characteristics of the musical stimuli

| Song name (condition) | *Envelope M±SD (a.u.)* | *Envelope Range (A.u.)* |
| --- | --- | --- |
| Hopp (Music) | 0.162±0.047 | -0.014-0.290 |
| Hopp (Shuffled Music) | 0.161±0.054 | -0.012-0.318 |
| Hopp  (High Pitch) | 0.160±0.046 | -0.011-0.290 |
| Hopp  (Low Pitch) | 0.180±0.046 | -0.007-0.317 |
| Lola  (Music) | 0.147±0.067 | -0.013-0.265 |
| Lola (Shuffled Music) | 0.154±0.059 | -0.005-0.305 |
| Lola  (High Pitch) | 0.144±0.067 | -0.013-0.265 |
| Lola  (Low Pitch) | 0.160±0.069 | -0.011-0.262 |

*Note*. *Hopp indicates the Hungarian playsong (“Hopp Juliska”), Lola indicates the Spanish playsong (“La vaca lola”).*
