# Supplementary material for: Development of auditory and spontaneous movement responses to music over the first postnatal year
Source: eLife. 2026 Jul 7;14:RP107088. doi: 10.7554/eLife.107088 (PMC13341111; doi:10.7554/eLife.107088)
Supplement: Peer review history

# Editor report: eLife Assessment

**DOI:** 10.7554/eLife.107088.4.sa0

**Authors:** Jessica Dubois

**Affiliations:** Inserm Unité NeuroDiderot, Université Paris Cité France

**Keywords:** Compelling, Important

This well-designed study offers **important** insights into the development of infants' responses to music based on the exploration of EEG neural auditory responses and video-based movement analysis. The **compelling** results revealed that evoked responses emerge between 3 and 12 months of age, but no age group demonstrated evidence of coordinated movements to music. This study will be of significant interest to developmental psychologists and neuroscientists, as well as researchers interested in music processing and in the translation of perception into action.

# Referee report: Reviewer #1 (Public review)

**DOI:** 10.7554/eLife.107088.4.sa1

Summary:

This study aims to investigate the development of infants' responses to music by examining neural activity via EEG and spontaneous body kinematics using video-based analysis. The authors also explore the role of musical pitch in eliciting neural and motor responses, comparing infants at 3, 6, and 12 months of age.

Strengths:

A key strength of the study lies in its analysis of body kinematics and modeling of stimulus-motor coupling, demonstrating how the amplitude envelope of music predicts infant movement, and how higher musical pitch may enhance auditory-motor synchronization.

EEG data provide evidence for enhanced neural responses to music compared to shuffled auditory sequences. These findings ecourage further investigation of the proposed developmental trajectory of neural responses to music and their link to musical behavior in infants.

Comments on revisions:

The authors have addressed my questions in their revision. I have no other questions. Thank you for the opportunity to read and evaluate this interesting study and also for all the work carried out in response to the comments.

# Author comment: Author response

**DOI:** 10.7554/eLife.107088.4.sa2

**Authors:** Trinh Nguyen, Félix Bigand, Susanne Reisner, Atesh Koul, Roberta Bianco, Gabriela Markova, Stefanie Hoehl, Giacomo Novembre

**Affiliations:** Italian Institute of Technology Rome Italy; Italian Institute of Technology Rome Italy; University of Vienna Vienna Austria; Italian Institute of Technology Genova Italy; Italian Institute of Technology Rome Italy; Paracelsus Medical University Salzburg Germany; University of Vienna Vienna Austria; Italian Institute of Technology Rome Italy

The following is the authors’ response to the previous reviews

**Public Reviews:**

**Reviewer #1 (Public review):**

Summary:

This study aims to investigate the development of infants' responses to music by examining neural activity via EEG and spontaneous body kinematics using video-based analysis. The authors also explore the role of musical pitch in eliciting neural and motor responses, comparing infants at 3, 6, and 12 months of age.

Strengths:

A key strength of the study lies in its analysis of body kinematics and modeling of stimulus-motor coupling, demonstrating how the amplitude envelope of music predicts infant movement, and how higher musical pitch may enhance auditory-motor synchronization.

EEG data provide evidence for enhanced neural responses to music compared to shuffled auditory sequences. These findings ecourage further investigation of the proposed developmental trajectory of neural responses to music and their link to musical behavior in infants.

Comments on revisions:

I thank the authors for the considerable effort devoted to revising the manuscript and addressing the raised questions and comments. I particularly appreciate the additional analyses and the extended arguments included in the discussion. I believe that this paper represents a valuable contribution to the literature on music development.

One remaining comment concerns the evoked response observed in the shuffled condition, which I still find intriguing. Considering that the auditory events in the shuffled condition display a clear rise time, particularly for those events that were selected based on being preceded and followed by longer periods of silence, one would expect to observe an evoked response emerging from baseline. However, this pattern is not evident in the presented curves. The authors may further examine and discuss the shape and characteristics of these response patterns.

We thank the Reviewer for highlighting this intriguing aspect of our data. We entirely agree that from a purely bottom-up, acoustic perspective, one would expect a clear onset-locked evoked response, such as an P1/P2 complex in adults or its developmental equivalent, given the prominent acoustic rise times and the surrounding periods of silence (such as those accounted for in the control analyses)

The fact that these responses are not present in the curves for the shuffled condition was striking to us as well. We interpret this severe attenuation not as a failure of sensory perception, but potentially as a consequence of higher-level cognitive modulation. Specifically, because the shuffled condition completely lacks structural regularities, the brain might be unable to build reliable temporal and/or melodic expectations. In the absence of a learnable structure, the auditory system likely down-weights the processing of these random sequences to conserve cognitive resources, leading participants to attentionally disengage.

This phenomenon aligns with both developmental and adult models of auditory processing. For instance, the "Goldilocks effect" demonstrates that infants systematically withdraw attention from auditory sequences that are entirely unpredictable (Kidd et al., 2014). Similarly, adult auditory literature suggests that while predictable patterns automatically capture attention, random and unpredictable acoustic streams could be actively tuned out (Dayan et al., 2000; Esber & Haselgrove, 2011).

Following the Reviewer’s helpful suggestion to further discuss the characteristics of these response patterns, we have expanded our description and interpretation of the shuffled condition curves in the revised manuscript. We added the following text to the Methods and Discussion to explicitly address the dampened shape of these responses:

p. 9: “Importantly, and in line with the adults’ data, all infant groups exhibited enhanced P1 amplitudes in response to music compared to shuffled music. Actually, across all groups, shuffled music did not elicit clear ERPs as the ones elicited by music”.

p.20: “This process was markedly dampened or interrupted by shuffled music (Bianco et al., 2024, 2025; Lense et al., 2022), a finding that could be interpreted as evidence of disengagement from such highly unpredictable sequences (Dayan et al., 2000; Esber & Haselgrove, 2011; Kidd et al., 2014).”

**Reviewer #2 (Public review):**

Summary:

Infants' auditory brain responses reveal processing of music (clearly different from shuffled music patterns) from the age of 3 months; however, they do not show related increase in spontaneous movement activity to music until the age of 12 months.

Strengths:

This is a nice paper, well designed, with sophisticated analyses and presenting clear results filling an important gap about early infant sensitivity, detection, and differentiation of musical sounds. The addition of EEG recordings (specifically ERPs) in response to music presentations at 3 different infant ages in the first postnatal year is important, and the manipulation of the music stimuli into shuffled, high and low pitch to capture differences in brain response processing and spontaneous movements is interesting. Further, the movement analysis based on Quantity of Movements (QoM) and movement subdivision into 10 distinct Principal Movements (PMs) is novel and creative.

Overall, results show that ERPs responses to music occurs earlier than QoM in early development, and that even at 12 months, motor responses to music remain coarse and not rhythmically aligned with the music tempo. This work increases our fundamental understanding of infants' early music perception in relation to auditory processing and motor response.

Comments on revisions:

The authors have addressed my questions in their revision. I have no other questions. Thanks again for the opportunity to read and evaluate this interesting work.

We thank the Reviewer for their time, their positive evaluation of our revised manuscript, and their constructive feedback throughout the review process, which has greatly helped us to strengthen this paper.

**Reviewer #3 (Public review):**

Summary

This study provides a detailed investigation of neural auditory responses and spontaneous movements in infants listening to music. Analyses of EEG data (event-related potentials and steady-state responses) first highlighted that infants at 3, 6 and 12 months of age and adults showed enhanced auditory responses to music than shuffled music. 6-month-olds also exhibited enhanced P1 response to high-pitch vs low-pitch stimuli, but not the other groups. Besides, whole body spontaneous movements of infants were decomposed into 10 principal components. Kinematic analyses revealed that the quantity of movement was higher in response to music than shuffled music only at 12 months of age. Although Granger causality analysis suggested that infants' movement was related to the music intensity changes, particularly in the high-pitch condition, infants did not exhibit phase-locked movement responses to musical events, and the low movement periodicity was not coordinated with music.

Strengths

This study investigates an important topic on the development of music perception and translation to action and danse. It targets a crucial developmental period that is difficult to explore. It evaluates two modalities by measuring neural auditory responses and kinematics, while cross-modal development is rarely evaluated. Overall, the study fills a clear gap in the literature.

Besides, the study uses state-of-the-art analyses. Detailed investigations were performed, as well as exploratory analyses in supplementary information. The discussion is rich in neurodevelopmental interpretations and comparisons with the literature. All steps are clearly detailed. The manuscript is very clear, well-written and pleasant to read. Figures are well-designed and informative. The authors' responses to previous reviews are also detailed and informative.

Comments on revisions:

The authors answered all my questions.

Thank you very much for your positive evaluation and for taking the time to review our revisions. We deeply appreciate your insightful comments across the review rounds, which have helped us improve the clarity and rigor of our paper.
